# Understanding near-surface polymer dynamics by a combination of grazing-incidence neutron scattering and virtual experiments

**DOI:** 10.1107/S1600576720014739

**Published:** 2021-02-01

**Authors:** Tetyana Kyrey, Marina Ganeva, Judith Witte, Regine von Klitzing, Stefan Wellert, Olaf Holderer

**Affiliations:** a Jülich Centre for Neutron Science at Heinz Maier-Leibnitz Zentrum, Forschungszentrum Jülich GmbH, Garching, Germany; b Institute of Chemistry, Technical University Berlin, Berlin, Germany; c Department of Physics, Technical University Darmstadt, Darmstadt, Germany

**Keywords:** grazing-incidence neutron spin-echo spectroscopy (GINSES), *BornAgain*, polymer dynamics, poly(*N*-isopropyl acrylamide) (PNIPAM), poly(ethylene glycol) (PEG)

## Abstract

Neutron spin-echo spectroscopy is a unique experimental method for the investigation of polymer dynamics. The combination of neutron spin-echo spectroscopy with grazing-incidence geometry (GINSES) opens the possibility to probe the dynamics of soft-matter materials in the vicinity of the solid substrate in the time range up to 100 ns. However, the usage of the GINSES technique has some peculiarities and, due to the novelty of the method and complexity of the scattering geometry, difficulties in further data analysis occur. The current work discusses how virtual experiments within the distorted-wave Born approximation using the *BornAgain* software can improve GINSES data treatment and aid the understanding of polymer dynamics in the vicinity of the solid surface. With two examples, poly(*N*-isopropyl acrylamide) brushes and poly(ethylene glycol) microgels on Si surfaces, the simulation as well as the application of the simulation to the GINSES data analysis are presented. The approach allowed a deeper insight to be gained of the background effect and scattering contribution of different layers.

## Abstract 2

Near-surface polymer dynamics are investigated with grazing-incidence neutron scattering and *BornAgain* virtual experiments.

## 1. Introduction   

Grazing-incidence neutron spin-echo spectroscopy (GINSES) is a powerful technique permitting investigation of the inner dynamics of various soft-matter systems at solid interfaces such as thin polymer films, layered microemulsions, phospholipid membranes *etc*. in the vicinity of the solid surface or buried interfaces (Nylander *et al.*, 2017 ▸; Jaksch *et al.*, 2015 ▸; Lipfert *et al.*, 2014 ▸; Frielinghaus, Kerscher *et al.*, 2012 ▸; Gawlitza *et al.*, 2015 ▸). Classical neutron spin-echo spectroscopy (Mezei, 1980 ▸; Ewen *et al.*, 1997 ▸) has the highest energy resolution of all spectroscopic techniques in neutron scattering and allows one to probe the thermal fluctuations or internal motions of polymers or proteins (Biehl *et al.*, 2008 ▸; Holderer *et al.*, 2008 ▸; Witte *et al.*, 2019 ▸). Unique properties of neutrons such as the ability to distinguish isotopes or light elements like H, D or C and low absorption for soft-matter systems (Hammouda, 2009 ▸) give direct access to the internal features of polymer systems. GINSES combines classical neutron spin-echo spectroscopy with the grazing-incidence geometry.

**Figure 1:**
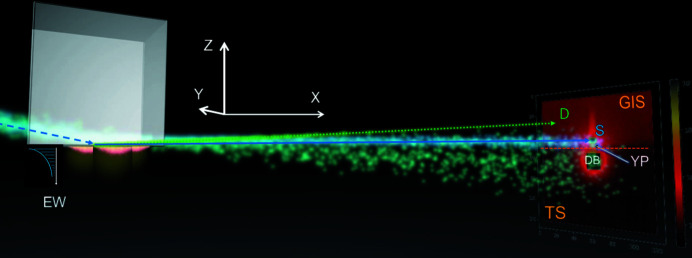
General illustration of the neutron scattering under grazing-incidence conditions. S – specular, D – diffuse scattering. The orange dashed line on the detector divides the scattering pattern into two regions: GIS, where grazing-incidence scattering is detected, and TS, where signal from transmitted scattering and the direct beam is detected. The white arrow on the 2D detector indicates the Yoneda peak (YP) and DB is the direct beam. The direct beam is usually blocked by a beamstop to avoid detector damage (black square). See the animation in the supporting information (this is a video illustration of the principles of grazing-incidence scattering).

In GINSES experiments, an approach similar to other techniques using grazing-incidence geometry [*e.g.* grazing-incidence small-angle neutron scattering (Wolff *et al.*, 2014 ▸; Oberdisse & Hellweg, 2017 ▸)] is applied. For soft-matter systems, the sample (microgels, brushes *etc*.) is typically adsorbed onto a silicon block and is illuminated from the substrate side with the neutron beam. In Fig. 1 ▸ an incoming beam (the blue dashed arrow) impinges onto the sample surface at a shallow incident angle below the critical angle of total reflection (Müller-Buschbaum, 2016 ▸). A 2D detector measures the specular and diffuse scattering intensity as a function of the exit angle and the out-of-plane angle. The graphics on the left indicated with ‘EW’ represent the propagation of the evanescent wave traveling parallel to the surface with a penetration depth of 10–100 nm perpendicular to the surface and an exponential decay of the intensity within the probed system. Under these conditions, near-surface dynamics can be probed. Moreover, by the variation of the scattering depth of the evanescent waves, *i.e.* variation of the incident angle, the dynamics as a function of distance to the interface can be studied (Müller-Buschbaum, 2013 ▸; Milosevic, 2013 ▸; Nouhi *et al.*, 2017 ▸). Thus, GINSES provides unique access to the internal dynamics of soft-matter systems at solid interfaces and is sensitive to the dynamics occurring in the pico- to nanosecond range (Holderer *et al.*, 2014 ▸; Jaksch *et al.*, 2019 ▸). However, performing the experiments and in particular the data treatment are still challenging tasks.

In real experiments, the incoming beam is not ideal, *i.e.* angular divergence, collimation limits and wavelength distribution are present. The latter complicates the estimation of the depth at which scattering occurs (scattering depth). There exists not a specific value but rather a range of scattering depths due to the experimental conditions and sample characteristics. For instance, the J-NSE spectrometer operated by Jülich Forschungszentrum at Maier-Leibnitz Zentrum (Garching, Germany) (Holderer *et al.*, 2008 ▸; Pasini *et al.*, 2019 ▸) has a wavelength resolution of 20%. At the SNS-NSE spectrometer at the Spallation Neutron Source (Oak Ridge, USA) (Ohl *et al.*, 2012 ▸) a chopper system selects wavebands of 3 Å. To correct the difference in the scattering depth, caused by a difference in wavelengths within the incoming pulse, application of a neutron prism was proposed and tested by Frielinghaus, Holderer *et al.* (2012 ▸).

In grazing-incidence geometry almost the whole incoming beam undergoes total reflection and the scattered intensity is usually weak, compared with the transmission geometry. To enhance this signal the application of a resonator waveguide was proposed. Initial experiments with one- and threefold resonators showed an increase of the scattering intensity by a factor of 3–7 (Kyrey, Witte *et al.*, 2018 ▸; Frielinghaus *et al.*, 2017 ▸, 2018 ▸).

Although the mentioned devices improve the enhancement of the signal-to-noise ratio in a GINSES experiment, the further data treatment and understanding are not trivial. There are still a number of open questions which are relevant for correct data analysis and interpretation, such as how to perform background correction, estimate the contributions from the different sample components to the total scattered intensity (block surface, additional layers as initiator or polyelectrolytes, used for growth or deposition of the samples onto solid surfaces), account for the instrument resolution, determine the scattering depth (which part of the sample is being probed) and so on. Such questions are also relevant for other surface-sensitive techniques such as neutron reflectometry (Hoogerheide *et al.*, 2020 ▸) and grazing-incidence small-angle scattering (Nouhi *et al.*, 2017 ▸). Therefore, in the present work, we demonstrate how these challenges can be tackled using simulations based on the distorted-wave Born approximation for GINSES.

In the present paper we address the challenges by means of simulation performed with the *BornAgain* software package (Pospelov *et al.*, 2020 ▸). This allows for simultaneous consideration of the structural features of the system of interest as well as instrumental characteristics (*e.g.* wavelength distribution, experimental setup). The application of the results ensuing from the virtual experiment for the further analysis of the polymer dynamics in the vicinity of a solid interface is discussed for the example of poly(ethylene glycol) (PEG)-based microgels and poly(*N*-isopropyl acrylamide) (PNIPAM) brushes adsorbed/grafted onto a silicon surface. Based on the previous investigations of the structural properties of these systems by means of atomic force microscopy, ellipsometry, neutron reflectometry and grazing-incidence small-angle neutron scattering (Gawlitza *et al.*, 2015 ▸; Wellert *et al.*, 2015 ▸; Witte *et al.*, 2020 ▸; Kyrey, Ganeva *et al.*, 2018 ▸), the application of the model that shows the best agreement with the real system is demonstrated. In contrast to layered microemulsions or diblock co-polymer systems, which possess a high contrast between scattering components (polymers with distinguishable scattering length densities or polymer/water walls), samples with low contrast with respect to the environment and a usually weak scattering signal are presented. We demonstrate that the dynamics of such polymer systems can be studied and characterized in the vicinity of the solid surface while analysis and dynamics characterization is improved by virtual experiments. The background contribution and information from different parts of the sample which would otherwise be inaccessible can be extracted in this way.

## 2. Materials and methods   

The structure of the PEG microgels and the PNIPAM brush used in the current work was previously studied by means of atomic force microscopy (AFM), neutron reflectometry (NR) and (grazing-incidence) small-angle neutron scattering (GISANS/SANS). The parameters applied for the model development are in agreement with the previous investigations (Wellert *et al.*, 2015 ▸; Gawlitza *et al.*, 2014 ▸; Witte *et al.*, 2020 ▸). The simulations take into account the instrument resolution, the layer roughness and the absorption contribution of each component. The simulated geometry of the experiment corresponds to the geometry of a real experiment (neutron beam penetrates sample through the Si block).

### 2.1. PEG microgel sample and model   

The microgels were synthesized by precipitation polymerization and consist of the monomer 2-(2-methoxy­ethoxy)ethyl methacrylate, the co-monomer poly(ethylene glycol)methyl ether methacrylate and the crosslinker ethylene glycol dimethacrylate. Details of sample preparation are given by Gawlitza *et al.* (2015 ▸). The system presented here has 5 mol% of co-monomer and the crosslinker amount was set to 3 mol%. The sample is designated as PEG microgel. This example was chosen since the preparation of layers of these microgels does not require any precursor layer to support the microgel–interface interaction. Hence, modeling of this example is based on a simple geometry.

**Figure 2:**
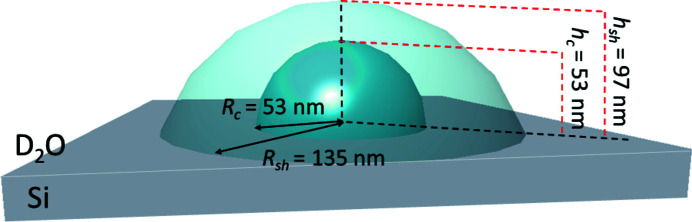
Schematic illustration of the PEG microgel model used for simulation. The microgel image was created with the *BornAgain* graphical user interface.

According to the previous structure investigation (Gawlitza *et al.*, 2014 ▸, 2015 ▸), the PEG microgel possesses a core–shell structure. Moreover, due to the adsorption onto a solid surface the spherical shape of the microgels undergoes a deformation. Using the microgel parameters from the previous SANS and AFM experiments, the PEG microgel was modeled as presented in Fig. 2 ▸: a core–shell particle with radii of 53 and 135 nm and heights of 53 and 97 nm for the core and the shell part, respectively. The form factor of a truncated sphere was applied.

To account for the effect of the density fluctuations, the form factor is represented as a sum of the form factors due to the scattering from the microgel shape 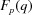 and the Ornstein–Zernike term related to the internal thermal fluctuations,
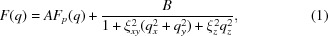
where *A* and *B* are the scaling factors for each of the contributions; 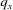, 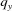 and 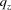 are the components of the scattering vector **q**; and 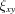, 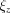 are the correlation lengths in the lateral and vertical directions, respectively. Since the microgel dimensions are sufficiently larger than the correlation length of internal density fluctuations, the cross-terms between both contributions can be neglected (Förster & Burger, 1998 ▸).

The scattering length densities (SLDs) for the shell of 3.9 × 10^−6^ Å^−2^ and for the core of 2.0 × 10^−6^ Å^−2^ are fixed according to the fitting result of NR data.

The Si/D_2_O interface roughness amplitude is fixed at 1.2 nm. A Hurst parameter of 0.8 was used in the roughness function to characterize the smoothness of the surface (Teichert *et al.*, 1995 ▸). The Hurst parameter (or Hurst exponent, or self-affine roughness exponent) characterizes self-affinity of the rough interface. It is constrained to be in the range between 0 and 1. Larger values are characteristic for smooth interfaces while smaller values occur for rough surfaces. More details on this parameter can be found elsewhere (Pospelov *et al.*, 2020 ▸; Chow, 2000 ▸). The lateral correlation length of the Si/D_2_O interface roughness was set to 570 nm. These parameters were obtained from NR and AFM data.

### 2.2. PNIPAM brush sample and model   

**Figure 3:**
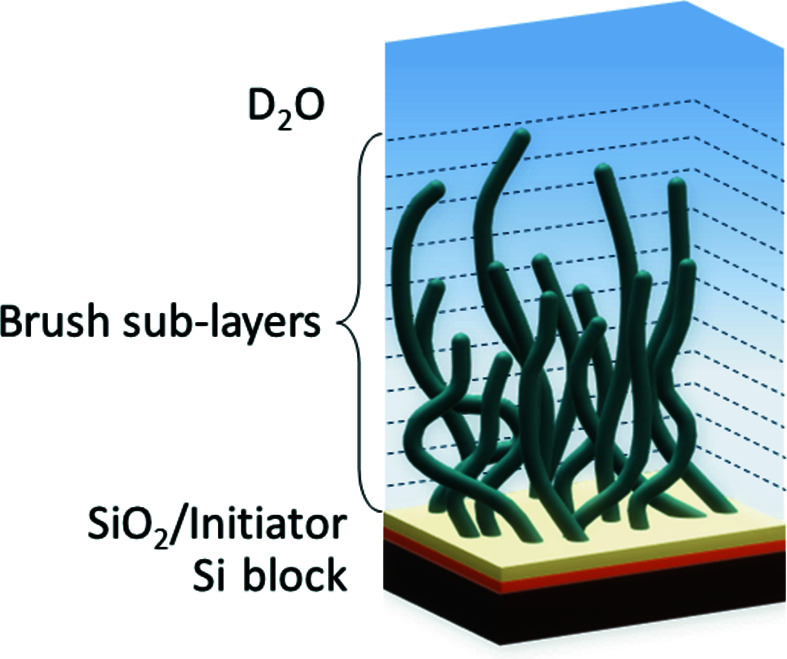
Schematic illustration of PNIPAM brush model used for simulation.

The PNIPAM brush discussed here was grafted onto silicon substrates by atom transfer radical polymerization as described in our previous work (Witte *et al.*, 2020 ▸). According to the reported results, the PNIPAM brush shows a gradual change of the polymer fraction along the surface normal, namely a lower water content in the vicinity of the solid surface and a gradually increasing amount of water in the outer brush region. Thus, the PNIPAM brush was modeled as one layer, placed between the quasi-infinite sub-layers of Si and D_2_O. A gradual SLD transition from polymer to heavy-water layer was simulated as a sequence of 100 sub-layers with a gradual SLD changing from 3.63 × 10^−6^ to 6.34 × 10^−6^ Å^−2^ in accordance with the self-consistent field theory (Milner *et al.*, 1988 ▸) as presented in equation (2):
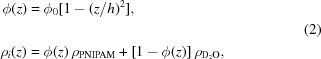
where *z* is the distance from the surface, 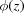 is the polymer volume fraction at *z*, 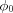 = 0.78 is the polymer volume fraction at 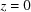, *h* is the thickness of the brush, and 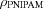 = 0.8 × 10^−6^ Å^−2^ and 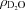 = 6.34 × 10^−6^ Å^−2^ are the SLDs of PNIPAM and D_2_O, respectively. The initiator layer used for the grafting of the PNIPAM brush on the silicon surface as well as the SiO_2_ layer were also accounted for in the model. To estimate background scattering, the Si block was modeled as a sub-layer with an SLD of 2.07 × 10^−6^ Å^−2^ and a roughness of 1 nm. According to Teichert *et al.* (1995 ▸), a Hurst parameter of 0.7 and a correlation parameter of 1 µm were applied. All parameters are listed in Table 1 ▸. In Fig. 3 ▸ a schematic illustration of the applied model is presented.

### 2.3. Simulation   

All simulations presented in the current work were performed in the framework of the distorted-wave Born approximation with the *BornAgain* software package (Pospelov *et al.*, 2020 ▸). *BornAgain* is an open-source multi-platform framework for simulation and fitting of grazing-incidence small-angle scattering and reflectometry data.

As mentioned above, the variation of the incident angle allows a variation of scattering depth, and thus the dynamics of different polymer layers can be probed. However, an estimation of the scattering depth as well as the distribution of the scattering intensities within the sample of interest is not trivial. Therefore, in this work, a 3D map of the intensity distribution as a function of the incidence angle and the scattering depth using the *BornAgain* software package was created. Instead of calculating the scattering depth for each particular combination of incident and scattering angles (Gawlitza *et al.*, 2015 ▸; Frielinghaus, Holderer *et al.*, 2012 ▸), the proposed approach allows one to simulate the full intensity map 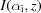 including specific sample characteristics (*e.g.* complex internal density distribution) as well as experimental peculiarities (*e.g.* wavelength distribution, beam divergence *etc*.). The intensity map was simulated as
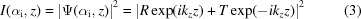
where Ψ is the amplitude of the neutron wave at the detector, 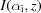 is the simulated intensity, which strongly depends on the incident angle (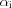) and scattering depth (*z*), 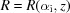 and 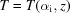 are the reflection and transmission coefficients, respectively, and 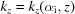 is the *z* component of the wavevector. In this case, the intensity at 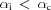 corresponds to the intensity of the evanescent wave, while the region at 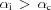 corresponds to the transmitted signal. The formalism implemented in *BornAgain* allows calculation of *R* and *T* parameters for each particular case. The unique SLD profile of each system is taken into account in the simulation procedure and, thus, the unique intensity map in accordance with the polymer system features can be obtained.

To obtain information about the evanescent wave intensity distribution, the intensity map for each system was simulated according to equation (3) and [Disp-formula fd3]on the basis of the models described above. Since the intensity maps are used for the further GINSES data analysis, the wavelength distribution of the J-NSE instrument of 20% (Pasini *et al.*, 2019 ▸) was applied.

The contribution of the background scattering to the general scattering signal was estimated via simulation of the 3D grazing-incidence small-angle scattering (GISAS) pattern as 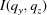. Intensity cuts along 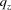 at 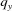 = 0 were analyzed. Intensities are not in absolute units as is common for SANS experiments. The main goal is to assess, for example, background contributions compared with sample contributions, or the contributions from different interface layers.

Since only the relative intensity was of interest, the virtual instrument setup varied in some parameters, but was kept the same for all simulations. However, to simulate the intensity distribution within the probed systems, obtain a signal/background ratio maximally corresponding to the real distribution, and then apply such results to the analysis of the measured data, the following parameters were adjusted to the real setups of the GINSES experiment: incident angle, wavelength distribution and simulated *q* range.

## 3. Results and discussion   

### 3.1. Intensity distribution   

**Figure 4:**
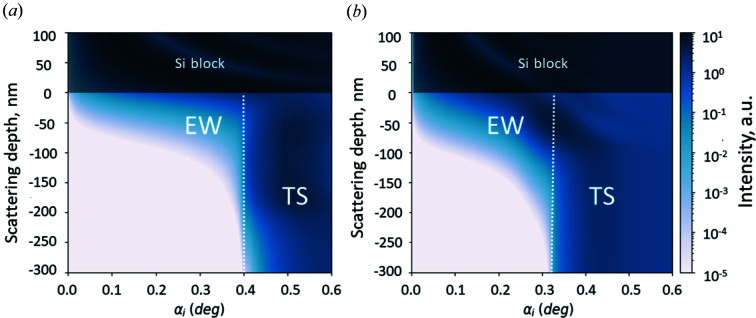
The distribution of the scattering intensity as a function of the incident angle (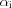) of the impinging neutrons and the distance from the scattering plane (scattering depth) for the PEG microgel (*a*) and PNIPAM brush (*b*) is mapped. Intensity is simulated according to equation (3). Zero marks the scattering plane; values above refer to positions inside the silicon subphase. The white dotted lines approximately separate evanescent wave (EW) and transmitted (TS) intensities.

On the basis of the presented models, the 3D intensity maps were simulated for the PEG microgel and the PNIPAM brush as presented in Fig. 4 ▸. The intensity in the region ‘Si block’ corresponds to the reflected signal. Intensity oscillations in this region are artificial and do not describe real intensity distributions. The intensity in the region marked as TS at 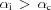 corresponds to the transmitted signal. The intensity in the region 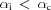 (marked as EW) corresponds to the evanescent wave intensity which exponentially decays away from the solid interface. It is clearly seen how an increase of the incident angle alters the penetration depth of the evanescent wave as well as its intensity distribution within the PEG microgel and the PNIPAM brush, namely an increase of the incident angle increases the penetration depth and thus deeper polymer layers can be probed. Moreover, the profile of the evanescent wave intensity distribution depends on the sample properties such as the SLD profile and interface roughness.

To estimate the scattering contribution from the different layers of the PEG microgel and the PNIPAM brush, cuts through the intensity map performed at constant incident angle were analyzed.

**Figure 5:**
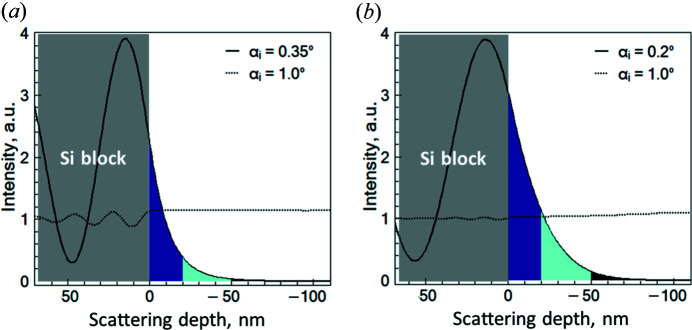
Intensity distribution within the PEG microgel (*a*) and PNIPAM brush (*b*) as a function of the scattering depth at the selected incident angles from Fig. 4 ▸: the solid lines at 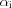 = 0.35° and 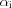 = 0.2°, respectively, correspond to the evanescent wave intensity at scattering depth < 0; the dotted lines at 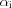 = 1.0° correspond to the transmitted signal. Blue, cyan and black blocks illustrate intensity at different scattering depth ranges (blue: 0–20 nm, cyan: 20–50 nm, black: >50 nm). The gray color region indicates the Si block.

In Fig. 5 ▸ the intensity as a function of the scattering depth at incident angles below and above the critical angle of total reflection is presented. The incident angles of 0.35 and 0.2° (below 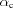) and the incident angle of 1.0° (above 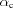) were initially chosen to experimentally probe the near-surface dynamics and the dynamics in the whole volume of the PEG microgel and PNIPAM brush with GINSES.

The colored areas in Fig. 5 ▸ represent the evanescent wave intensity distribution in different parts of the samples. From the quantitative analysis at 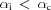, it can be concluded that 70% of the evanescent wave intensity falls on the first 20 nm of the PNIPAM brush, whereas in the case of the PEG microgel 82% of the evanescent wave intensity falls on the same thickness. Hence, the dynamics in this near-surface region strongly contribute to the experimentally measured intermediate scattering function 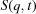. This should be considered in the comparison of the data from the different samples.

### 3.2. Background scattering   

In the simplest case, the system of interest consists of the sample (colloids, microgels, brushes *etc*.) and the chemically homogeneous substrate (*e.g.* Si, quartz glass). However, in a real experiment the sample may contain additional components such as a precursor layer or anchoring molecules (*e.g.* initiator molecules) supporting the adhesive contact between the sample itself and the substrate. Furthermore, the substrate may be chemically heterogeneous, *e.g.* due to the presence of oxide layers. These structural characteristics have to be considered in terms of the SLD profile and roughness. Additionally to the scattering from the PEG microgel and PNIPAM brush, the scattering from the solid surface, solvent and auxiliary layers (SiO_2_, initiator) contributes to the background scattering. To characterize the studied system, these contributions should be separated or accounted for during data analysis. Unfortunately, in a grazing-incidence scattering experiment a background measurement can not be directly subtracted from the total signal as in transmission experiments (*e.g.* SANS). Therefore, to estimate the contribution from the substrate and the different parts of the investigated systems to the measured scattering pattern, the simulation of GISAS with and without the polymer system was the only way to access background contributions. While only relative intensities were of interest, instrument parameters were selected and kept fixed for all simulations. The intensities were compared at *q* values of 0.08 and 0.06 Å^−1^ for the PEG microgel and PNIPAM brush, respectively (such a *q* value was chosen for the GINSES experiment due to the appropriate signal-to-noise ratio).

To correctly simulate the roughness of the Si block, the following parameters were applied: the Si block was modeled as a sub-layer with an SLD of 2.07 × 10^−6^ Å^−2^ and a roughness of 1 nm. Following Teichert *et al.* (1995 ▸), a Hurst parameter of 0.7 and a correlation parameter of 1 µm were applied.

**Figure 6:**
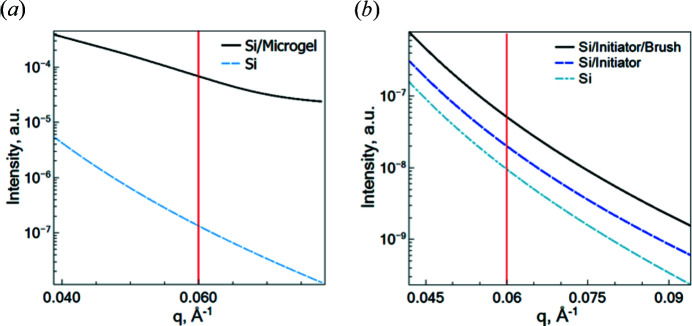
Scattering contribution of the Si block and the initiator layer to the general scattering intensity of the PEG microgel (*a*) and PNIPAM brush (*b*) simulated in the grazing-incidence geometry. All simulations are performed against D_2_O. The light-blue dashed–dotted line is the scattering signal from the neat Si block, the blue dashed line that from the Si block coated with the initiator layer (in the case of the PNIPAM brush), and the black solid line is the scattering signal from the corresponding polymer system onto the Si block. The red line indicates the *q* value at which GINSES measurements were performed.

According to Fig. 6 ▸(*b*) the intensity contribution of the Si block to the full scattering signal of the PNIPAM brush at *q* = 0.06 Å^−1^ is 19%, while the contribution from the Si block with an initiator layer is 40%. Note that the experimentally measured scattering intensity of the Si block against D_2_O is higher than the scattering from the grafted brushes. Such a difference could be explained by the difference in the roughness contribution. In grazing-incidence geometry roughness is one of the parameters leading to an imperfection of the scattering surface and, thus, causing the grazing-incidence diffuse scattering. Speculatively, the coating of the Si block with the initiator layer decreases the scattering contrast between Si and D_2_O and, therefore, the background contribution from the Si surface becomes weaker. As a consequence, the reference measurements in a GINSES experiment can not be used for direct estimation of the background contribution; however they allow estimation of additional contributions, *e.g.* due to instrumental imperfections.

In the case of the PEG microgel the simulated contribution from the Si block at *q* = 0.08 Å^−1^ is less than 1% [see Fig. 6 ▸(*a*)].

### 3.3. Application to the GINSES data   

**Figure 7:**
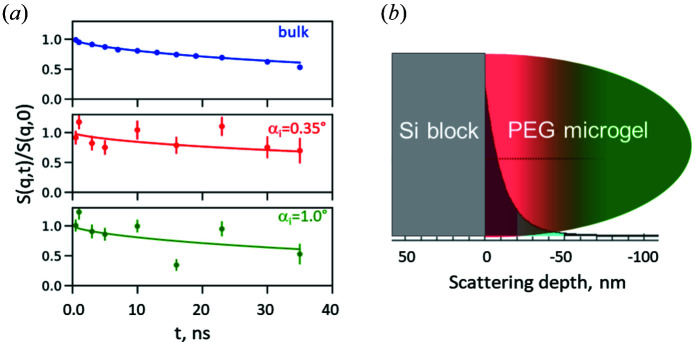
(*a*) Intermediate scattering functions of the PEG microgels measured in the bulk (blue) and adsorbed state at an incident angle of 0.35° (red) and 1.0° (green) (Gawlitza *et al.*, 2015 ▸). (*b*) Schematic illustration of the evanescent wave intensity distribution within a PEG microgel according to the simulation from Fig. 5 ▸. The red–green color transition indicates depths probed at different incident angles in grazing-incidence geometry; colors correspond to the data in (*a*). The shape of the red–green figure schematically represents the shape of the PEG microgel.

Fig. 7 ▸ shows the data from GINSES experiments on the PEG microgel in bulk and at the interface at *q* = 0.08 Å^−1^ [as reported by Gawlitza *et al.* (2015 ▸)] as well as a schematic illustration of the probed microgel regions at different incidence angles 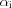. The red–green area represents scattering depths probed at incident angles of 0.35 and 1° and corresponds to the intermediate scattering functions (ISFs) measured at these angles. As background in Fig. 7 ▸(*b*) the simulated evanescent wave intensity distribution is shown. According to the measured ISF, the bulk sample and that in the adsorbed state probed at 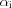 = 1.0° (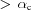) possess the same relaxation rate of 0.01 ns^−1^, while a reduction of the overall relaxation rate in the vicinity of the solid surface to 0.0036 ns^−1^ at 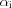 = 0.35°(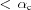) is observed. Such behavior can be explained by the performed simulation as follows.

According to Fig. 5 ▸(*a*), at 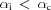 mostly the near-surface layers of the PEG microgel contribute to the scattering signal, namely the first 20 nm at the incident angle of 0.35°. The adsorption of the microgels onto a solid interface causes their compression and the formation of a more densely packed polymer sub-layer close to the Si surface occurs. A similar tendency was earlier found for PNIPAM microgels (Kyrey *et al.*, 2019 ▸). This causes the limitation of the polymer chain dynamics and leads to the slowing of the relaxation rate. Thus, instead of fitting the relaxation rate for 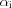 = 0.35° with about 20 nm scattering depth separately, the fit function applied to the ISF has been split into a bulk-like and a rigid component (as an extreme case) having the formula
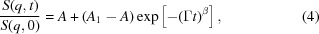
with an elastic component *A* and amplitude 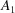, and with a relaxation rate Γ, a Fourier time *t* measured with neutron spin-echo (NSE) and a stretching exponent β. Bulk data are background corrected and the ISF starts at 1, which is not the case for the GINSES measurements, since background subtraction is not feasible in the usual way. It is the aim of this paper to obtain reliable background contributions from the *BornAgain* simulations. GINSES data have been normalized to 1 in Fig. 7 ▸ to make it easier to compare with the bulk measurement, by dividing equation (4) by 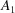. Faster processes than those accessible with NSE may be present in the sample but are excluded from analysis, in bulk NSE measurements by background subtraction, and in GINSES by this normalization to 1. The relaxation rate Γ = 0.01 ns^−1^ was taken from the bulk measurement; β = 0.67 as in the work of Gawlitza *et al.* (2015 ▸). A background contribution from the Si block of 1% was estimated from the simulation. Such a value is in the range of error and does not have an influence on data fitting.

The obtained ratio 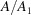 = 0.17 indicates that at least 1/6 of the material in the first 20 nm of the particle has a reduced mobility compared with the bulk (assuming here mobility zero, otherwise it would be a larger fraction).

At 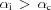, on the other hand, the relaxation rate value measured in grazing-incidence geometry is equal to that obtained in the transmission neutron spin-echo experiment, namely 0.01 ns^−1^ at 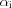 = 1° and *q* = 0.8 Å^−1^, as mentioned previously.

At first sight, this is counter-intuitive, since the confinement region (close to the substrate) should noticeably contribute to the scattering and reduce the relaxation rate. However, the above simulation allows one to estimate the contribution of the different layers of the probed system to the scattering signal at 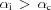. According to the intensity cut at an incident angle of 1° presented in Fig. 5 ▸(*a*), different microgel layers of the same thickness contribute to the general signal equally. Taking into account the ratio of the thickness of the first dense layer and the rest of the microgel of approximately 1:4 and the evanescent wave intensity distribution according to the performed simulation, the scattering contribution from the first layers (20 nm) becomes significantly smaller than that from the rest.

Moreover, the adsorption process similar to the temperature collapse of the polymer chains leads to a decrease of the internal scattering contrast. This leads also to a SANS intensity decrease and to a different quasielastic contribution to the GINSES signal at the probed *q*. From the SANS data of the PEG microgel the scattering intensity ratio at *q* = 0.08 Å^−1^ in the swollen and collapsed state is approximately 2:1 (Gawlitza *et al.*, 2014 ▸). This additionally decreases the contribution from the near-surface layer. It means that at 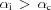 the outer bulk-like region to a large extent contributes to the general scattering signal, which explains the equal values of the relaxation rate of the microgel in bulk and in the adsorbed state when the whole sample thickness is probed.

In summary, contributions to the intermediate scattering function could be separated into a ‘bulk-like’ (soft) relaxation with amplitude (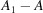) of the ISF for parts of the microgel far from the interface, and a ‘rigid’ contribution with amplitude *A* of the ISF close to the interface (which in reality could be less ‘binary’ but with a gradient from one to the other, but this is clearly beyond the available data precision). This separation of contributions instead of a single relaxation as used in the previous evaluation is useful in combination with *BornAgain* simulations of the intensity. These allow one to assign a length scale to the layer with reduced mobility, which would be very difficult to impossible at interfaces with less intuitive SLD variations compared with thin straight layers.

This example illustrates that the *BornAgain* simulations can help to obtain a deeper understanding of the interface properties even if the statistics do not justify the fit, *e.g.* stretch exponents as in a standard bulk experiment. Instead of including all changes into a changed relaxation rate, an immobile fraction could be extracted.

**Figure 8:**
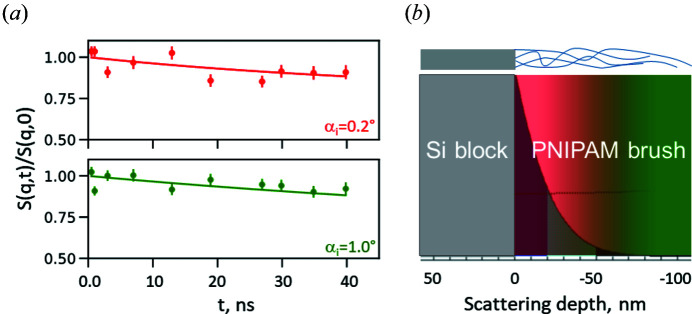
(*a*) Intermediate scattering functions of the PNIPAM brush measured in the grafted state at an incident angle of 0.2° (red) and 1.0° (green). Data were normalized according to the fitting amplitude. (*b*) Schematic illustration of the evanescent wave intensity distribution within a PNIPAM brush. The red–green color transition indicates the depths probed at different incident angles in grazing-incidence geometry; colors correspond to the data in (*a*).

As a second example, the dynamics of the PNIPAM brush measured with GINSES have been analyzed in a similar way with equation (4). Fig. 8 ▸ represents the ISFs of a PNIPAM brush collected at incident angles of 0.2 and 1° as well as the distribution of the evanescent wave within the sample according to the simulation presented in Fig. 5 ▸. Here, data were normalized to 1 as in the previous example by dividing 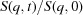 by the fitted amplitude, since we can not apply the usual background subtraction procedure.

The bulk relaxation rate of a PNIPAM solution at *q* = 0.06 Å^−1^ is Γ = 0.022 ns^−1^ from the work of Witte *et al.* (2019 ▸) with β = 1 since this dense brush layer is assumed to be still in the density fluctuation regime (Witte *et al.*, 2020 ▸). The near-surface dynamics measured with GINSES are very close to the bulk value (Γ = 0.019 ns^−1^) with a ratio 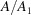 = 0.75. This means only 1/4 of the scattering signal contributes to fluctuations.

*BornAgain* simulations can not only provide information on the penetration depth of the evanescent wave in rather complex samples but also help in the assessment of the background contribution, if the virtual experiment reflects realistically the true experimental conditions. The virtual experiment allows one then to remove parts of the sample and observe the remaining scattering contribution at otherwise exactly identical conditions. In the real world, this would require separate sample preparations on different Si blocks.

According to the simulation presented in Fig. 6 ▸, the background contribution from the Si block with the initiator layer is about 40%; with *A* = 0.6 this would result in a background fraction of 0.24. The remaining part of 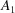, 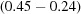 = 0.21, can then be attributed to an immobilized (in the probed time range) part of the brush close to the interface. This experiment has been performed at 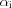 = 1° and 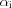 = 0.2°, probing the full brush height in the first case and approximately the first 25% of the layer in the second case. Witte *et al.* (2020 ▸) attributed this to an increase in dynamic correlation length with increasing distance from the interface.

Thus, *BornAgain* simulations help here to separate the contribution from ‘background scattering’ and ‘sample scattering’. Moreover, the combination of scattering techniques with the *BornAgain* simulations allows one to extract a dependence of the relaxation rates on distance from the surface and to estimate the influence of a substrate on the inner dynamics of a soft sample in contact with this substrate.

## 4. Conclusion   

In the present work, it is demonstrated how virtual experiments performed with *BornAgain* improve a grazing-incidence scattering data analysis for two examples of a PEG microgel and a PNIPAM brush.

Based on the previous studies of these systems, corresponding models considering sample inhomogeneities such as internal density fluctuation or a smooth polymer/D_2_O transition were developed. The *BornAgain* modeling approach allows one to work with complex systems and to represent them with a maximal chosen and automatically generated number of layers with variable thickness and SLD profile.

On the basis of the performed modeling and simulations, the polymer dynamics of PEG microgel and PNIPAM brush have been analyzed with GINSES. The fraction of the scattering contribution from the polymer system was determined after extraction of the background contribution obtained with the virtual experiment, since standard background determination is impossible in GINSES experiments. The background fraction in the whole scattering amplitude of 0.24 was estimated for the PNIPAM brush. In the case of the PEG microgel, the virtual experiment allowed us to describe the dynamics of the polymer layers even at low statistics of the scattering signal. It was estimated that 1/6 of the near-surface layer possesses a reduced mobility compared with the dynamics in the bulk state.

The results presented illustrate that virtual experiments can contribute to a better understanding of the near-surface behavior of complex polymer systems (even with low contrast with respect to the environment or low scattering intensity and/or low-ordered structure).

## Supplementary Material

Video illustration of principles of grazing-incidence scattering. DOI: 10.1107/S1600576720014739/ge5086sup1.mov

## Figures and Tables

**Table 1:** Fitting parameters of PNIPAM brush according to the initial one-layer model

System layer	Thickness (nm)	SLD × 10^−6^ (Å^−2^)	Roughness (nm)
D_2_O	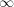	6.34	–
PNIPAM brush	81	3.63–6.34 (100 slices)	–
Initiator	1.3	0.56	<1
SiO_2_	1.3	3.47	<1
Si block	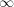	2.07	<1
